# Correction: Investigation of synovial fluid induced *Staphylococcus aureus* aggregate development and its impact on surface attachment and biofilm formation

**DOI:** 10.1371/journal.pone.0233534

**Published:** 2020-05-14

**Authors:** Matthew J. Pestrak, Tripti Thapa Gupta, Devendra H. Dusane, Doug V. Guzior, Amelia Staats, Jan Harro, Alexander R. Horswill, Paul Stoodley

The following information is missing from the Funding statement: ARH is supported by NIH public health service grant AI083211.
