# Supplementary material for: Investigation of synovial fluid induced Staphylococcus aureus aggregate development and its impact on surface attachment and biofilm formation
Source: PLoS One. 2020 Apr 17;15(4):e0231791. doi: 10.1371/journal.pone.0231791 (PMC7164621; doi:10.1371/journal.pone.0231791)
Supplement: Attachment — Submitted filename: PLos One Response to Reviewers (1).docx [file pone.0231791.s002.docx]

Dear Dr. Abdelwahab Omri,

Please find uploaded our revised manuscript, PONE-D-20-01923 “Investigation of synovial fluid induced *Staphylococcus aureus* aggregate development and its impact on surface attachment and biofilm formation.”

We have responded to each of the critiques outlined by the reviewers. We found the reviews to

be accurate and helpful in the development of this manuscript and sincerely appreciate the

constructive comments, which have helped to clarify several important points in the manuscript

and present the data more clearly and concisely to the readers. We trust that the corrections

made to the manuscript make it acceptable for publication.

Below is an item-by-item response to the critique:

**Reviewer #1**

*Comment:* Only a few minor - recommendations and these all relate to the need for bacterial names to be italicized in the reference list. References 14, 20 and 40 all have bacterial names that have not been italicized.

**Response:** The bacterial names in the references have been italicized.

**Reviewer #2:**

*Comment:* *The authors cite dated literature in the Introduction, which could be markedly improved. Current data on the incidence and costs of PJI have recently been reported [1], and there is new consensus information of biofilm formation in PJI [2].*

**Response:** We thank the reviewer for this correction, and we have included these references and updated the text accordingly (see Lines 59, 61, & 76)

*Comment:* *While the authors’ focused study on synovial fluid aggregates and implant biofilm formation is justified, it is now known that S. aureus utilizes four distinct mechanisms of biofilm formation during PJI [3]. Thus, the lack of studies on Staphylococcus abscess communities (SACs) and colonization of osteocytic canalicular networks in cortical bone need to be acknowledged as limitations.*

**Response:** We agree with the reviewer that this is a limitation of our study, and discussion of this limitation has been included in the discussion (see Lines 335-339).

*Comment: The absence of in vivo studies should also be acknowledged as a limitation.*

**Response:** We also agree that this is another limitation of the study, and discussion of this has been included in the discussion (see Lines 344-346).

*Comment:* *The authors conclude that the aggregation threshold of 60% in their studies is due to a size limit or an upper limit of detection with their method. However, this could also be due to Agr mediated emigration.*

**Response:** We agree that this is another possible explanation for our observations, and we have included this interpretation in the results section (see Lines 199-200).
